# Proteomic Analysis of Post-synaptic Density Fractions from *Shank3* Mutant Mice Reveals Brain Region Specific Changes Relevant to Autism Spectrum Disorder

**DOI:** 10.3389/fnmol.2017.00026

**Published:** 2017-02-14

**Authors:** Dominik Reim, Ute Distler, Sonja Halbedl, Chiara Verpelli, Carlo Sala, Juergen Bockmann, Stefan Tenzer, Tobias M. Boeckers, Michael J. Schmeisser

**Affiliations:** ^1^Institute for Anatomy and Cell Biology, Ulm UniversityUlm, Germany; ^2^International Graduate School in Molecular Medicine, Ulm UniversityUlm, Germany; ^3^Institute for Immunology, University Medical Center of the Johannes-Gutenberg University MainzMainz, Germany; ^4^Focus Program Translational Neurosciences, University Medical Center of the Johannes-Gutenberg University MainzMainz, Germany; ^5^CNR Neuroscience InstituteMilan, Italy; ^6^BIOMETRA, University of MilanMilan, Italy; ^7^Division of Neuroanatomy, Institute of Anatomy, Otto-von-Guericke UniversityMagdeburg, Germany; ^8^Leibniz Institute for NeurobiologyMagdeburg, Germany

**Keywords:** Shank3, autism spectrum disorder, synapse, proteome, striatum, Homer1

## Abstract

Disruption of the human *SHANK3* gene can cause several neuropsychiatric disease entities including Phelan-McDermid syndrome, autism spectrum disorder (ASD), and intellectual disability. Although, a wide array of neurobiological studies strongly supports a major role for SHANK3 in organizing the post-synaptic protein scaffold, the molecular processes at synapses of individuals harboring *SHANK3* mutations are still far from being understood. In this study, we biochemically isolated the post-synaptic density (PSD) fraction from striatum and hippocampus of adult *Shank3Δ11^-/-^* mutant mice and performed ion-mobility enhanced data-independent label-free LC–MS/MS to obtain the corresponding PSD proteomes (Data are available via ProteomeXchange with identifier PXD005192). This unbiased approach to identify molecular disturbances at *Shank3* mutant PSDs revealed hitherto unknown brain region specific alterations including a striatal decrease of several molecules encoded by ASD susceptibility genes such as the serine/threonine kinase Cdkl5 and the potassium channel K_Ca_1.1. Being the first comprehensive analysis of brain region specific PSD proteomes from a *Shank3* mutant line, our study provides crucial information on molecular alterations that could foster translational treatment studies for *SHANK3* mutation-associated synaptopathies and possibly also ASD in general.

## Introduction

Studies from the last decade have repeatedly outlined that genetic disruptions of *SHANK3* in humans are of upmost clinical relevance as they can lead to various neuropsychiatric disorders including the PMS, a complex neurodevelopmental condition and syndromic autism variant, non-syndromic ASD and ID (Durand et al., 2007; Betancur and Buxbaum, 2013; Kleijer et al., 2014; Leblond et al., 2014). As *SHANK3* encodes a large scaffold protein organizing the PSD of excitatory glutamatergic synapses (Boeckers, 2006) it is hypothesized that *SHANK3* mutations perturb synaptic transmission in neural circuits throughout the brain and thereby cause diverse neuropsychiatric phenotypes (Grabrucker et al., 2011). To identify the respective circuits and the underlying molecular pathomechanisms, several *Shank3* mutant mouse lines have been engineered up to this date (Bozdagi et al., 2010; Peca et al., 2011; Wang et al., 2011, 2016; Schmeisser et al., 2012; Han et al., 2013; Kouser et al., 2013; Lee J. et al., 2015; Speed et al., 2015; Bidinosti et al., 2016; Jaramillo et al., 2016a,b; Mei et al., 2016; Zhou et al., 2016). Importantly, mutants from each line exhibit behavioral traits related to neuropsychiatric diseases and neurobiological alterations at the synaptic level that could be of further use for the development of targeted therapies (Jiang and Ehlers, 2013; Schmeisser, 2015). However, most of the studies have thus far included the molecular analysis of a biased selection of synaptic proteins that had previously been identified in Shank3 interaction studies, i.e., direct binding partners such as GKAP/SAPAP or Homer and indirect binding partners such as MAGUKs or glutamate receptors (Boeckers, 2006). Additionally, previous studies mainly focused on a single brain region and most of them analyzed crude synaptosomes rather than purified PSD fractions. However, for a better understanding of the synaptic pathology of *SHANK3* mutations in neuropsychiatric diseases, we need an unbiased and more comprehensive insight into the molecular PSD composition of *Shank3* mutant mice in ASD-associated brain regions. For this reason, we biochemically isolated the PSD fraction from striatum and hippocampus of *Shank3Δ11^-/-^* deletion mutants, performed ion-mobility enhanced DIA label-free LC–MS/MS to obtain the corresponding PSD proteomes. These data are not only essential to better understand the molecular anatomy of PSDs devoid of major Shank3 isoforms, but will also help to foster translational treatment studies for *SHANK3* mutation-associated synaptopathies in the future.

## Materials and Methods

### Mice

Generation of *Shank3Δ11^-/-^* mutant mice has been described previously (Schmeisser et al., 2012). Mice were bred on a C57BL/6J background and housed under standard laboratory conditions (average temperature of 22°C, food and water available *ad libitum*). Lights were automatically turned on/off in a 12 h rhythm. All homozygous *Shank3Δ11^-/-^* mutants and WT littermates used for this study were from hetero-hetero breedings, from both sexes and between P56 and P84. Animal experiments were approved by the review board of the Land Baden-Wuerttemberg, Permit Number 0.103 and performed in compliance with the guidelines for the welfare of experimental animals issued by the Federal Government of Germany.

### Antibodies

The anti-Shank3 antibody has been described previously (Schmeisser et al., 2012). The other antibodies have been obtained from commercial suppliers as it follows: anti-PSD95 (dilution: 1:2 000, Synaptic Systems, #124011, RRID:AB_10804286), anti-Synaptophysin (dilution: 1:20 000, Abcam, Cambridge, UK, #ab14692, RRID:AB_301417), anti-rabbit HRP (dilution: 1:1 000, Dako, Hamburg, Germany, #P0399, RRID:AB_2617141), and anti-mouse HRP (dilution: 1:3 000, Dako, Hamburg, Germany, #P0260, **RRID** not available).

### Subcellular Fractionation and Western Blot

Subcellular fractionation of brain tissue was performed as previously described with minor modifications (Distler et al., 2014b). For each of the *n* = 5 samples per genotype used for proteomic analysis, striata or hippocampi of five individual mice were combined. All steps were performed at 4°C. Tissue was homogenized (Ho) in buffer A (0.32 M sucrose, 5 mM HEPES, pH 7.4) and centrifuged at 1 000 × *g*. The supernatant (S1) was further centrifuged at 12 000 × *g* and the pellet containing the crude membrane fraction (P2) was obtained. This fraction was solubilized in buffer B (0.32 M sucrose, 5 mM Tris-HCl, pH 8.1) and loaded on a discontinuous sucrose step gradient (0.85 M/1.0 M/1.2 M). After centrifugation at 85 000 × *g* the synaptosomes (Syn) were collected from the 1.0 M/1.2 M interface and incubated with buffer C (0.32 M sucrose, 12 mM Tris, pH 8.1, 1% Triton X-100). After centrifugation at 32 800 × *g*, the PSD pellet was collected (PSD) and solubilized in H_2_O.

Equal amounts of 10 μg from each sample were separated via SDS-PAGE and subsequently blotted on Nitrocellulose membranes according to standard protocols. Incubation with the primary antibody was followed by incubation with an HRP-conjugated secondary antibody (Dako). Signals were visualized with the Pierce ECL Western Blotting Substrate and further detected with the MicroChemi 4.2 machine. Signals were quantified with Gelanalyzer software ^1^.

### Tryptic Digestion

Aliquots corresponding to 20 μg PSD protein were digested using a modified filter-aided sample preparation (FASP) protocol, which has been previously described in detail (Wisniewski et al., 2009; Distler et al., 2016). Prior to LC–MS analysis, the resulting tryptic digest solutions were diluted to a concentration of 500 ng/μL using aqueous 0.1% formic acid and spiked with 25 fmol/μL of enolase 1 (*Saccharomyces cerevisiae*) tryptic digest standard (Waters GmbH, Eschborn, Germany).

### Nanoscale Liquid Chromatography Mass Spectrometry (nanoLC–MS) of Tryptic Digests

NanoLC–MS analysis of tryptic peptides was performed as described before using a nanoAcquity UPLC system (Waters) coupled to a Waters Synapt G2-S HDMS mass spectrometer (Waters) (Distler et al., 2014b, 2016). In brief, peptides were separated using a HSS-T3 C18 1.8 μm, 75 μm × 250 mm reversed phase column. Mobile phase A was water containing 0.1% formic acid and 3% DMSO. Mobile phase B was ACN containing 0.1% formic acid and 3% DMSO. Samples were loaded onto the column in direct injection mode with 1% mobile phase B as described before (Distler et al., 2014b, 2016). Peptides were separated using a gradient from 1 to 35% mobile phase B over 90 min at a flow rate of 300 nL/min. After separation of peptides, the column was rinsed with 90% mobile phase B, followed by a re-equilibration step at initial conditions (1% mobile phase B) resulting in a total run time of 120 min. [Glu1]-fibrinopeptide was used as lock mass at 100 fmol/μl. All samples were analyzed in three technical replicates.

NanoESI-MS analysis of tryptic peptides on the Waters Synapt G2-S system was performed in positive V-mode with a resolving power of at least 25 000 FWHM (full width half maximum). The instrument was equipped with a NanoLockSpray source and the lock mass channel was sampled every 30 s. LC–MS data were collected using ion mobility enhanced MS^E^ (Silva et al., 2005; Geromanos et al., 2012). Precursor ion information was collected in low-energy MS mode applying a constant collision energy of 4 eV. Fragment ion information was obtained in the elevated energy scan using drift-time specific collision energies as detailed before (UDMS^E^) (Distler et al., 2014a, 2016). The spectral acquisition time in each mode was 0.6 s with a 0.05 s-interscan delay resulting in an overall cycle time of 1.3 s for the acquisition of one cycle of low and elevated energy data.

### Raw Data Processing and Data Analysis

Initial signal processing of continuum LC–IMS-MS^E^ data and subsequent database search were performed using vendor software ProteinLynx Global SERVER (PLGS, version 3.02, Waters). For protein and peptide identification, data were searched against a custom compiled database containing UniProtKB/Swiss-Prot entries of the mouse reference proteome (UniProtKB release 2014_02, 16 780 entries). Sequence information for enolase 1 (*Saccharomyces cerevisia*e) as well as for common contaminants (i.e., human keratins, porcine trypsin) was added to the databases. Following criteria were applied for database search: (i) trypsin as digestion enzyme, (ii) up to two missed cleavages per peptide, (iii) a minimum peptide length of six amino acids, (iv) carbamidomethyl cysteine as fixed and (v) methionine oxidation as variable modification. The false discovery rate (FDR) for peptide and protein identification was assessed searching a reverse database and set to 0.01 for database search in PLGS.

Label-free quantification analysis was performed using the ISOQuant software tool (Distler et al., 2014a, 2016). Briefly, the analysis included retention time alignment, exact mass retention time (EMRT) and IMS clustering as well as data normalization and protein homology filtering. Settings and algorithms have been described in detail in previous protocols (Distler et al., 2014a, 2016). The peptide-level FDR for cluster annotation was set to 0.01 within ISOQuant. For further quantitative analysis, only proteins identified by at least two peptides with a minimum length of six amino acids were considered. Additionally, to be included in the final dataset, a protein had to be identified in at least four biological replicates in at least one condition (i.e., either in WT striatum, *Shank3Δ11^-/-^* mutant striatum, WT hippocampus or *Shank3Δ11^-/-^* mutant hippocampus). For each protein, absolute in-sample amounts were estimated using TOP3 quantification (Silva et al., 2006). Based on the quantitative information derived from the TOP3 approach, we calculated the relative amount of each protein with respect to the sum over all detected proteins [ppm: parts per million (w/w) of total protein]. The mass spectrometry proteomics data have been deposited to the ProteomeXchange Consortium^2^ via the PRIDE partner repository (Vizcaino et al., 2014, 2016) with the dataset identifier PXD005192.

Statistical analysis of the data was conducted using Student’s *t*-test with Bonferroni correction for multiple hypothesis testing to largely exclude potential false-positive hits. As this is a very conservative and stringent approach, Bonferroni adjusted *p*-values of *p* ≤ 0.05 were considered statistically significant to not exclude proteins that are actually displaying differences between WT and *Shank3Δ11^-/-^* mutants. In addition, for each protein we calculated the log-transformed ratio of its average amount in WT mice divided by its average amount in *Shank3Δ11^-/-^* mutant mice for hippocampus and striatum. To be included in the list of regulated proteins, proteins had to be statistically significant (*p* ≤ 0.05) and display a log_2_ ratio of at least ±0.24 between WT and *Shank3Δ11^-/-^* mutant mice.

Functional annotation and analysis of proteins that displayed significant changes between WT and *Shank3Δ11^-/-^* mutant mice was performed using DAVID Bioinformatics Resources (version 6.7,)^3^ (Huang da et al., 2009a,b). To identify autism-associated gene products among the pool of changed proteins in the *Shank3Δ11^-/-^* mutant PSD from either brain region, proteins were individually matched with the SFARI (Simons Foundation Autism Research Initiative) autism gene database^4^ (Banerjee-Basu and Packer, 2010). For the evaluation of known and predicted protein–protein interactions among these proteins, the Search Tool for the Retrieval of Interacting Genes/Proteins (STRING) database v10.0^5^ was used (Szklarczyk et al., 2015). Protein–protein interactions were visualized with the Gephi software v0.9.1^6^ (Bastian et al., 2009).

## Results

### Proteomic Characterization of the Striatal and Hippocampal PSD from *Shank3Δ11^-/-^* Mutants

**FIGURE 1:**
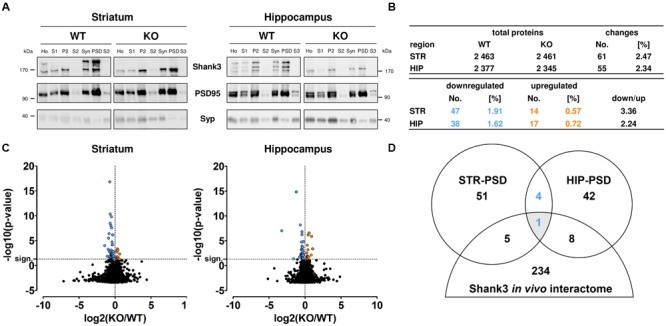
**Large-scale proteomic analysis of wild type (WT) and *Shank3Δ11^-/-^* mutant (KO) post-synaptic density (PSD) fractions from striatum and hippocampus. (A)** Western Blot analysis of the subcellular fractions derived from PSD isolation: Homogenate (Ho), Purified homogenate (S1), Crude membrane fraction (P2), Cytosol (S2), Synaptosomes (Syn), PSD and Synaptic cytosol (S3) from WT and *Shank3Δ11^-/-^* mutant (KO) tissue. Note enrichment of Shank3 and PSD95 and depletion of Synaptophysin (Syp) in the PSD fraction of both, striatum (STR) and hippocampus (HIP). Representative bands for indicated proteins are shown. **(B)** Total number of proteins and the significantly changed proteins (blue: down-regulated; orange: up-regulated) identified in the PSD fraction of WT and *Shank3Δ11^-/-^* mutant (KO) striatum (STR) or hippocampus (HIP) as indicated. The remaining Shank3 protein is excluded from this analysis. **(C)** Volcano plots of all molecular alterations in the WT and *Shank3Δ11^-/-^* mutant (KO) PSD fraction from striatum and hippocampus [log_2_(KO/WT), x-axis] and the respective statistical significance [-log10(*p*-value), y-axis]. Significantly regulated proteins (above horizontal dashed line marked as “sign.”) are colored (blue and left of vertical dashed line: down-regulated; orange and right of vertical dashed line: up-regulated). Changes that did not reach statistical significance remained black. Statistical analysis was performed using a Bonferroni-corrected unpaired two-tailed *t*-test and a sample size of *n* = 5 independent biological replicates. **(D)** Venn Diagram showing the number of proteins with altered expression levels in the *Shank3Δ11^-/-^* mutant PSD from striatum (STR) and hippocampus (HIP) overlapping with a Shank3 *in vivo* interactome from synaptosomes (Han et al., 2013) (blue: down-regulated).

After subcellular fractionation, we performed Western Blot analyses to confirm the purity of the isolated PSD fractions. As expected, the anti-Shank3 antibody differentiated between material from WT and *Shank3Δ11^-/-^* mutant (KO) samples. Our analysis confirmed that the post-synaptic scaffold protein PSD95 was increased and the presynaptic vesicle protein Synaptophysin was decreased in the PSD fraction from each brain region of both genotypes when compared to the corresponding homogenate or synaptosomal fraction, respectively (**Figure 1A**; **Supplementary Figure S1**). In our proteomic analysis, we identified 2 461 proteins in the striatal and 2 345 proteins in the hippocampal PSD from *Shank3Δ11^-/-^* mutant animals using an ion-mobility enhanced DIA LC–MS approach (Distler et al., 2014a). Similar results were obtained from WT material (**Figure 1B**; Supplementary Tables S1 and S2; **Supplementary Figure S2**). Further analysis revealed that 61 proteins (2.47%) were significantly altered within the striatal and 55 (2.34%) within the hippocampal PSD from *Shank3Δ11^-/-^* mutant animals (p ≤ 0.05). In both brain regions, PSDs derived from *Shank3Δ11^-/-^* mutant animals displayed higher numbers of down- than up-regulated proteins (**Figures 1B,C**). All regulated proteins including KO/WT abundance ratios are listed in detail in Supplementary Table S2. Notably, Shank3 was still detectable in the *Shank3Δ11^-/-^* mutant in our proteomic analysis at ∼41% of WT levels in the striatal and ∼24% of WT levels in the hippocampal *Shank3Δ11^-/-^* mutant PSD. This result served as internal control due to the fact that the *Shank3Δ11^-/-^* mutants are devoid of major, but not all Shank3 isoforms (**Figure 1A**; Supplementary Tables S1 and S2; **Supplementary Figure S3**) (Schmeisser et al., 2012; Vicidomini et al., 2016; Wang et al., 2016). Biochemical analysis further revealed that the protein levels of the remaining isoforms, Shank3e and Shank3f, were not altered in the PSD fraction from either brain region (**Supplementary Figure S4**). We next analyzed if there were converging changes among the regulated proteins in the *Shank3Δ11^-/-^* mutant PSD from both brain areas when compared with the Shank3 *in vivo* interactome from murine synaptosomes (Han et al., 2013). Intriguingly, only one protein was identified (**Figure 1D**): Homer1, which was decreased in both the striatal and hippocampal PSD of *Shank3Δ11^-/-^* mutants (Supplementary Table S2).

### Gene Ontology Enrichment Analysis Reveals Distinct Changes of Biological Processes and Molecular Functions in Striatal and Hippocampal PSD Proteomes of *Shank3Δ11^-/-^* Mutants

**FIGURE 2:**
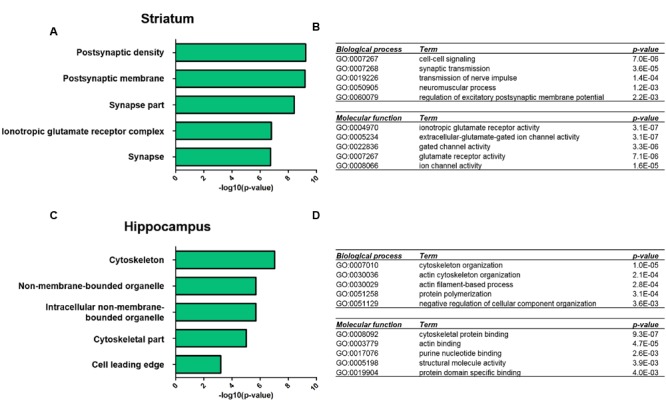
**Gene ontology analysis of significantly altered proteins in the *Shank3Δ11^-/-^* mutant PSD from striatum and hippocampus.** Functional annotation of proteins with significantly altered expression levels in the striatal **(A,B)** and hippocampal **(C,D)** *Shank3Δ11^-/-^* mutant PSD was performed using DAVID (v6.7, https://david.ncifcrf.gov) with the data in Supplementary Table S2 used as input. Bar diagrams visualize the top five significantly enriched GO terms for ‘cellular compartment’ for molecular alterations in the striatal **(A)** or hippocampal **(C)** PSD. The top five significantly enriched GO terms for ‘biological process’ and ‘molecular function’ for molecular alterations in the striatal **(B)** or hippocampal **(D)** PSD are listed in table-like diagrams.

We next performed GO-term based protein enrichment analysis to gain more insight into the functionality of the molecular changes in the striatal and hippocampal PSD proteome of *Shank3Δ11^-/-^* mutant animals. Statistical enrichment analysis of the three categories “cellular compartment,” “biological process,” and “molecular function” yielded distinct results among the two brain regions. In the striatal *Shank3Δ11^-/-^* mutant PSD, the top five enriched GO-terms from all three categories pointed toward altered levels of proteins exhibiting ionotropic glutamate receptor activity and involved in cell–cell signaling at the PSD as the most prominent molecular consequences of Shank3 deficiency (**Figures 2A,B**). For example, this included a decrease of the AMPAR subunits GluA1 and GluA2, the NMDAR subunits GluN1 and GluN2B and the kainate receptor GluK5 (Supplementary Table S2). Contrary to that, the same type of analysis revealed that in the hippocampal *Shank3Δ11^-/-^* mutant PSD proteins involved in cytoskeleton organization such as Abi1, Gelsolin or Profilin2 were predominantly changed following loss of Shank3 (**Figures 2C,D**; Supplementary Table S2).

### The Striatal PSD Proteome of *Shank3Δ11^-/-^* Mutants Comprises More Altered Proteins Encoded by ASD Susceptibility Genes than the Hippocampal One

**FIGURE 3:**
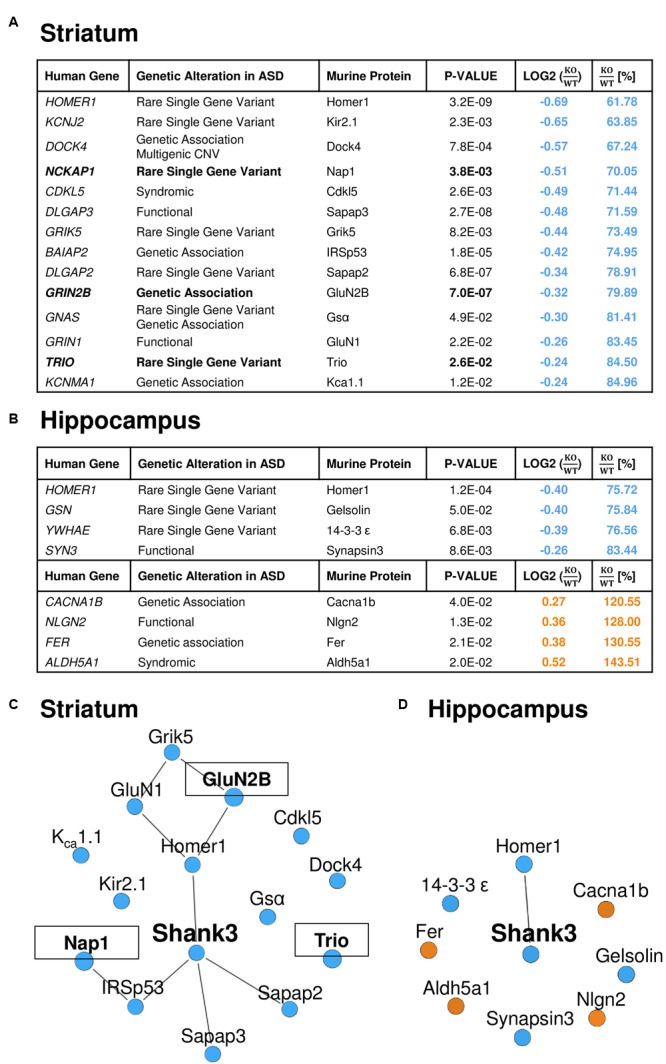
**A relevant number of the significantly altered proteins in the *Shank3Δ11^-/-^* mutant PSD from striatum is encoded by ASD susceptibility genes.** Significantly altered proteins in the striatal **(A)** and hippocampal **(B)** *Shank3Δ11^-/-^* mutant (KO) PSD match with entries of the SFARI autism gene database. Proteins further encoded by high-risk TADA genes are highlighted in bold. The KO/WT ratios are marked in blue for down- and in orange for up-regulated proteins. **(C,D)** Protein–protein interactions (lines) among the significantly altered proteins in the striatal **(C)** and hippocampal **(D)** *Shank3Δ11^-/-^* mutant PSD that match with entries in the SFARI autism gene database. Proteins, which are further encoded by TADA genes, are marked in bold and are in boxes. Proteins significantly down-regulated in *Shank3Δ11^-/-^* mutant mice are marked in blue, significantly up-regulated proteins in orange.

Similar to the GO-term based protein enrichment analysis, comparison of the quantitative datasets of altered proteins in the striatal or hippocampal PSD proteome of *Shank3Δ11^-/-^* mutant animals with the 826 autism-associated primary target genes presently listed in the SFARI autism gene database also rendered distinct results: 14 of the 61 proteins (23%) altered in the striatal and 8 of the 55 proteins (15%) altered in the hippocampal *Shank3Δ11^-/-^* mutant PSD matched with proteins encoded by SFARI autism genes (**Figures 3A,B**). The only converging molecular alteration in this context was a decrease of Homer1 in both *Shank3Δ11^-/-^* mutant PSD proteomes, while all other changes were unique to each brain region. Importantly, all molecules changed in the striatal *Shank3Δ11^-/-^* mutant PSD that matched with the SFARI autism gene database were reduced and comprised the murine homologs of proteins encoded by several major ASD candidates (**Figure 3A**) including *NCKAP1, GRIN2B* and *TRIO*, 3 of the 65 high-risk ASD TADA genes (De Rubeis et al., 2014; Sanders et al., 2015). Based on these results, we generated minimal distance interaction networks that showed a high degree of interactions among these molecules almost exclusively in the striatal *Shank3Δ11^-/-^* mutant PSD (**Figures 3C,D**). The core of this network comprises known direct and indirect Shank interactors (Han et al., 2013) and is extended by several other intriguing candidates that have not yet been associated with Shank3 or Shankopathies including the serine/threonine kinase Cdkl5 and the potassium channels Kir2.1 and K_Ca_1.1.

## Discussion

In this study, we used ion-mobility enhanced data-independent label-free LC–MS/MS to generate an unbiased dataset of *in vivo* changes in the PSD fraction from both striatum and hippocampus of adult *Shank3Δ11^-/-^* mutant mice lacking major isoforms of Shank3 (Schmeisser et al., 2012; Vicidomini et al., 2016). We have previously shown by the same methodological approach (biochemical isolation followed by LC–MS/MS) that in this fraction, PSD-specific proteins were highly enriched whereas contaminating proteins (i.e., mitochondrial or presynaptic ones) were efficiently decreased (Distler et al., 2014b).

Our comprehensive analysis quantified approximately 2 500 proteins in the striatal and 2 400 proteins in the hippocampal *Shank3Δ11^-/-^* mutant PSD and identified several significantly regulated proteins largely distinct for either brain region. These findings are intriguing as they point toward a specific function of Shank3 in organizing the molecular anatomy of the PSD in a brain region specific manner. Interestingly, our GO-term enrichment analysis revealed that deficiency of Shank3 in the striatal PSD primarily results in changes of proteins involved in glutamatergic synaptic transmission, while cytoskeleton-associated proteins were mainly affected in the *Shank3Δ11^-/-^* mutant hippocampal PSD. This is supported by previous studies on impaired striatal glutamatergic synaptic transmission in the same (Vicidomini et al., 2016) and other *Shank3* mutants (Peca et al., 2011; Jaramillo et al., 2016a,b; Peixoto et al., 2016; Wang et al., 2016; Zhou et al., 2016) and on impaired cytoskeletal organization in hippocampal neurons with altered gene dosage or protein structure of Shank3 (Durand et al., 2012; Han et al., 2013) In addition, loss of differential Shank3 isoforms with distinct functions from striatal or hippocampal PSDs in a varying degree depending on their physiological expression pattern and levels throughout the brain might play a role in this context (Wang et al., 2014).

Based on the fact that approximately 0.7% of individuals with ASD exhibit a mutation in *SHANK3* (Leblond et al., 2014), we further compared our datasets with the SFARI autism gene database to identify molecular patterns that could be of relevance for a better understanding of ASD-associated pathomechanisms in our model. Interestingly, we only found one protein to be altered in the *Shank3Δ11^-/-^* mutant PSD of both brain regions: Homer1. This post-synaptic scaffold protein and C-terminal Shank interactor, which interconnects the latter with group I metabotropic glutamate receptors and regulators of post-synaptic calcium signaling (Tu et al., 1999; Sala et al., 2001, 2005; Hayashi et al., 2009) was decreased predominantly in the striatal PSD. These data support previous biochemical findings in the same (Vicidomini et al., 2016) and other *Shank3* mutants (Peca et al., 2011; Wang et al., 2011, 2016; Jaramillo et al., 2016a,b; Zhou et al., 2016) pointing toward a common molecular phenotype at synapses caused by any genetic disruption of *Shank3*. Together with the fact that several rare variants of the *HOMER1* gene have been found in individuals with ASD (Kelleher et al., 2012) our data strongly underline the central role of *HOMER1* gene dosage and – as we have previously demonstrated – to the associated mGlu5 altered signaling (Vicidomini et al., 2016) to better understand the molecular underpinnings of *SHANK3* mutation-associated ASDs. This finding subsequently calls for a detailed analysis of ASD-like behaviors and the underlying molecular pathomechanisms in *Homer1* mutants. Intriguingly, we also found that almost 25% of the altered proteins in the striatal *Shank3Δ11^-/-^* mutant PSD matched with the SFARI autism gene database. These molecules were all reduced and comprised several major ASD candidates including three proteins encoded by the murine homologs of the TADA genes *NCKAP1, GRIN2B* and *TRIO*, which are highly relevant for ASD formation in humans (De Rubeis et al., 2014; Sanders et al., 2015). Minimal distance interaction network analysis further showed that the majority of these proteins interact with each other pointing toward a defined ASD-associated molecular network that is disrupted specifically at the PSD of *Shank3Δ11^-/-^* mutant corticostriatal synapses. The core of this network includes known direct and indirect Shank interactors including Homer1, IRSp53, members of the GKAP/SAPAP family and the NMDA receptor, a well-known target of translational pharmacotherapy of ASD-like phenotypes in mice (Grabrucker et al., 2011; Han et al., 2013; Jiang and Ehlers, 2013; Lee E.J. et al., 2015). In addition, the network comprises several other intriguing ASD-related molecules that have not yet been associated with Shank or Shankopathies and could well serve as targets for future treatment studies. Among these is Cdkl5, a serine/threonine kinase involved in Akt-mTOR signaling at the synapse, genetically related to several neurodevelopmental disorders in humans including ASD, Rett syndrome and epileptic encephalopathies and whose disruption leads to ASD-like behavior and impaired neural circuitry in mice (Wang et al., 2012; Sivilia et al., 2016). Other intriguing candidates are the potassium channels Kir2.1 and K_Ca_1.1. Especially the latter is of high interest as genetic disruption is not only found in individuals with ASD and epilepsy, but also impairs network properties in ASD-related brain regions in mice including Purkinje cell circuits (Guglielmi et al., 2015). Importantly, our data on the exclusive reduction of a significant number of ASD-associated molecules in the striatal PSD of *Shank3Δ11^-/-^* mutants mirror findings from previous studies on two different gene targeted mouse models of ASD: *Pten^m3m4^* mutants expressing the mistargeted tumor suppressor Pten and *Fmr1^-^*^/^*^-^* mutants lacking the fragile X mental retardation protein (Fmrp). Transcriptome analysis of *Pten^m3m4^* mutant cortices revealed broad down- and proteomic analysis of *Fmr1^-^*^/^*^-^* mutant cortical synaptosomes broad up-regulation of many human ASD-susceptibility genes (Tang et al., 2015; Tilot et al., 2016). We therefore again emphasize the need of unbiased and comprehensive screening of both gene expression and molecular synapse anatomy in ASD-associated brain regions of genetically based model systems to not only understand the molecular consequences of the corresponding mutation, but also the molecular pathology of ASD in a broader fashion.

## Author Contributions

MS, TB, and ST designed research. JB supervised mouse breeding. DR, UD, and SH carried out experiments; DR, UD, CV, CS, and ST performed data analysis. DR, UD, ST, and MS designed all figures. MS jointly wrote the manuscript with all other authors.

## Conflict of Interest Statement

The authors declare that the research was conducted in the absence of any commercial or financial relationships that could be construed as a potential conflict of interest.

## Footnotes

1 www.gelanalyzer.com/

2 http://proteomecentral.proteomexchange.org

3 https://david.ncifcrf.gov

4 https://gene.sfari.org/autdb/Welcome.do

5 http://string-db.org

6 https://gephi.org

## Supplementary Material

FIGURE S1 **Complete Western blot membranes. Complete membranes of the Western blots presented in **Figure 1**: (A,B)** Subcellular fractions derived from striatal **(A)** or hippocampal **(B)** PSD isolation as indicated: Homogenate (Ho), Purified homogenate (S1), Crude membrane fraction (P2), Cytosol (S2), Synaptosomes (Syn), PSD and Synaptic cytosol (S3) from WT and *Shank3Δ11^-/-^* mutant (KO) tissue. Signals for Shank3, PSD95 and Synaptophysin (Syp) are indicated. **(A,B)** Red boxes show the cutting lines for the blots used in **Figure 1**.

FIGURE S2 **Further characterization of the proteomic dataset. (A)** Dynamic range of detected PSD proteins in the striatum (STR) and hippocampus (HIP) of WT and *Shank3Δ11^-/-^* mutant (KO) mice. **(B)** Overlap of proteins identified in the PSDs derived from the striatum (STR) and hippocampus (HIP) of WT and *Shank3Δ11^-/-^* mutant (KO) mice. Only proteins that have been identified in at least four biological replicates in one condition (either STR WT, STR KO, HIP WT, or HIP KO) were considered. **(C)** Correlation plots of technical (upper panels) and biological (lower panels) replicates are exemplarily shown for each brain region in WT mice. The correlation plots show high reproducibility of sample preparation and high precision of label-free quantification between technical replicates as well as biological samples.

FIGURE S3 ***Shank3* genetic targeting strategy and resulting pattern of Shank3 isoforms still present in *Shank3Δ11^-/-^* mutant mice. (A)** Schematic illustration of the murine *Shank3* gene (boxes represent respective exons) showing the deletion of exon 11 (indicated in red) and the remaining Shank3 isoforms based on the review by Jiang and Ehlers (2013) excluding alternative splice variants (ANK: N-terminal ankyrin repeats; SH3: Src homology 3 domain; PDZ: PSD95/DLG/ZO-1 domain; Pro: Proline-rich clusters; SAM: sterile alpha motif). Theoretical molecular weight of each Shank3 isoform in kDa was calculated from the isoform specific exon coding sequences and is indicated. **(B)** Amino acid sequence of the longest Shank3 isoform Shank3a. Colors indicate the respective protein–protein interaction domains; dashed boxes represent the peptides identified by nanoLC–MS, light boxes: detection only in WT tissue, darker boxes filled in gray: detection in both WT and *Shank3* mutant tissue.

FIGURE S4 **Analysis of *Shank3* isoforms still present in *Shank3Δ11^-/-^* mutant mice. (A)** Schematic illustration of the murine *Shank3* gene (boxes represent respective exons) showing the deletion of exon 11 (indicated in red) and the remaining Shank3 isoforms based on the review by Jiang and Ehlers (2013) excluding alternative splice variants (ANK: N-terminal ankyrin repeats; SH3: Src homology 3 domain; PDZ: PSD95/DLG/ZO-1 domain; Pro: Proline-rich clusters; SAM: sterile alpha motif). Theoretical molecular weight of each Shank3 isoform in kDa was calculated from the isoform specific exon coding sequences and is indicated. The epitopes of the anti-Shank3 antibody used in this study are marked as red bars below the schematic illustration of the Shank3f isoform **(B,C)** Western blot analysis of striatal **(B)** and hippocampal **(C)** PSD fractions of WT and *Shank3Δ11^-/-^* mutant (KO) mice. Shank3 isoforms a, c, e, and f are indicated largely based on the study by Wang et al. (2014). Observed Shank3 isoforms appear with a shift of additional ∼60 kDa from their calculated molecular weight, most probably due to post-translational modifications. Right panel: Analysis of signal intensities for Shank3e and Shank3f, which are still present in the *Shank3Δ11^-/-^* mutant PSDs. No significant change was observed. Statistical analysis was performed using an unpaired, two-tailed *t*-test with a biological sample size of *n* = 3.
