# Supplementary figures and images for: Proteomic Analysis of Post-synaptic Density Fractions from Shank3 Mutant Mice Reveals Brain Region Specific Changes Relevant to Autism Spectrum Disorder
Source: Front Mol Neurosci. 2017 Feb 14;10:26. doi: 10.3389/fnmol.2017.00026 (PMC5306440; doi:10.3389/fnmol.2017.00026)

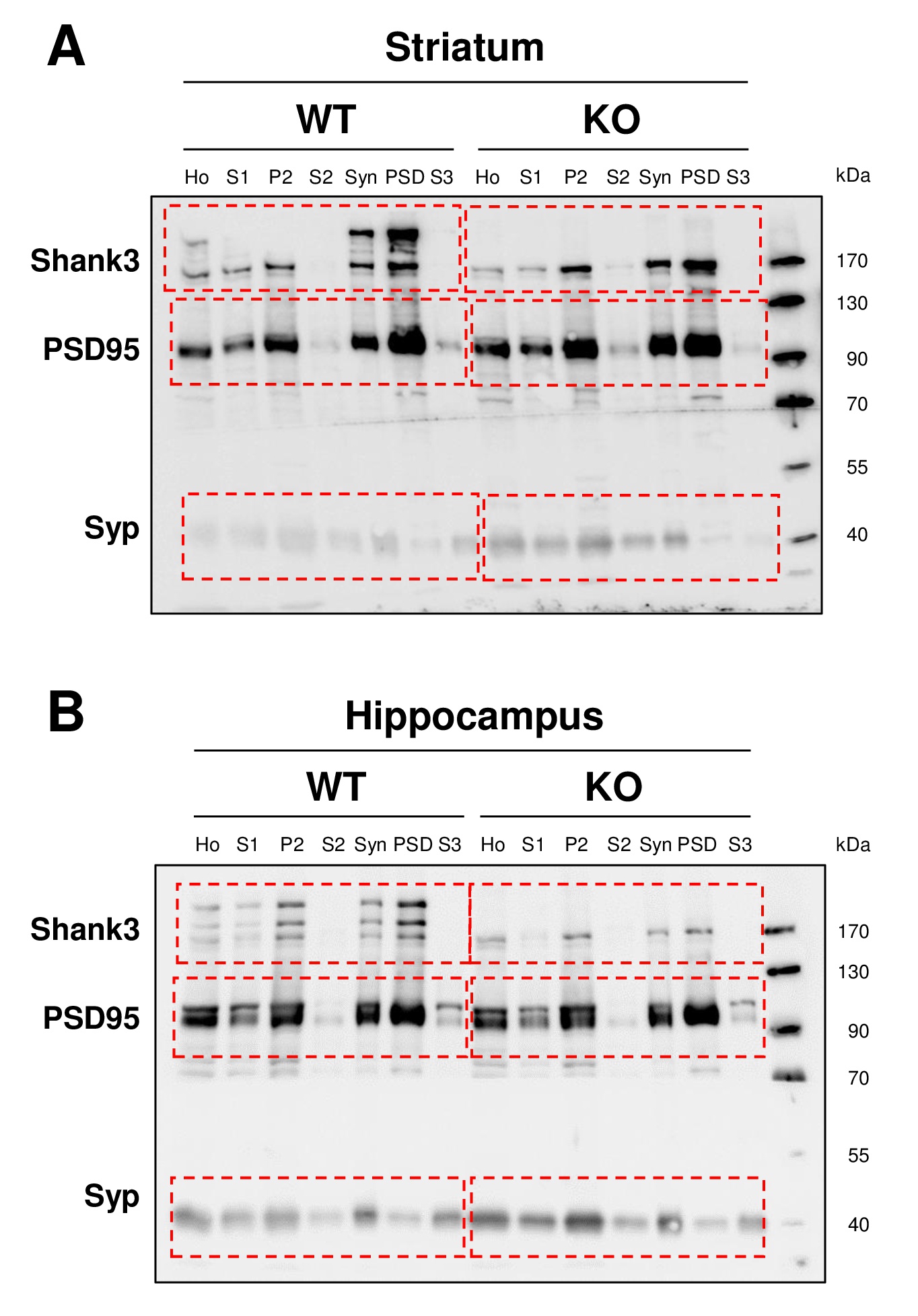

Supplement: FIGURE S1 — Complete Western blot membranes. Complete membranes of the Western blots presented in Figure 1: (A,B) Subcellular fractions derived from striatal (A) or hippocampal (B) PSD isolation as indicated: Homogenate (Ho), Purified homogenate (S1), Crude membrane fraction (P2), Cytosol (S2), Synaptosomes (Syn), PSD and Synaptic cytosol (S3) from WT and Shank3Δ11-/- mutant (KO) tissue. Signals for Shank3, PSD95 and Synaptophysin (Syp) are indicated. (A,B) Red boxes show the cutting lines for the blots used in Figure 1. [file Image_1.JPEG]

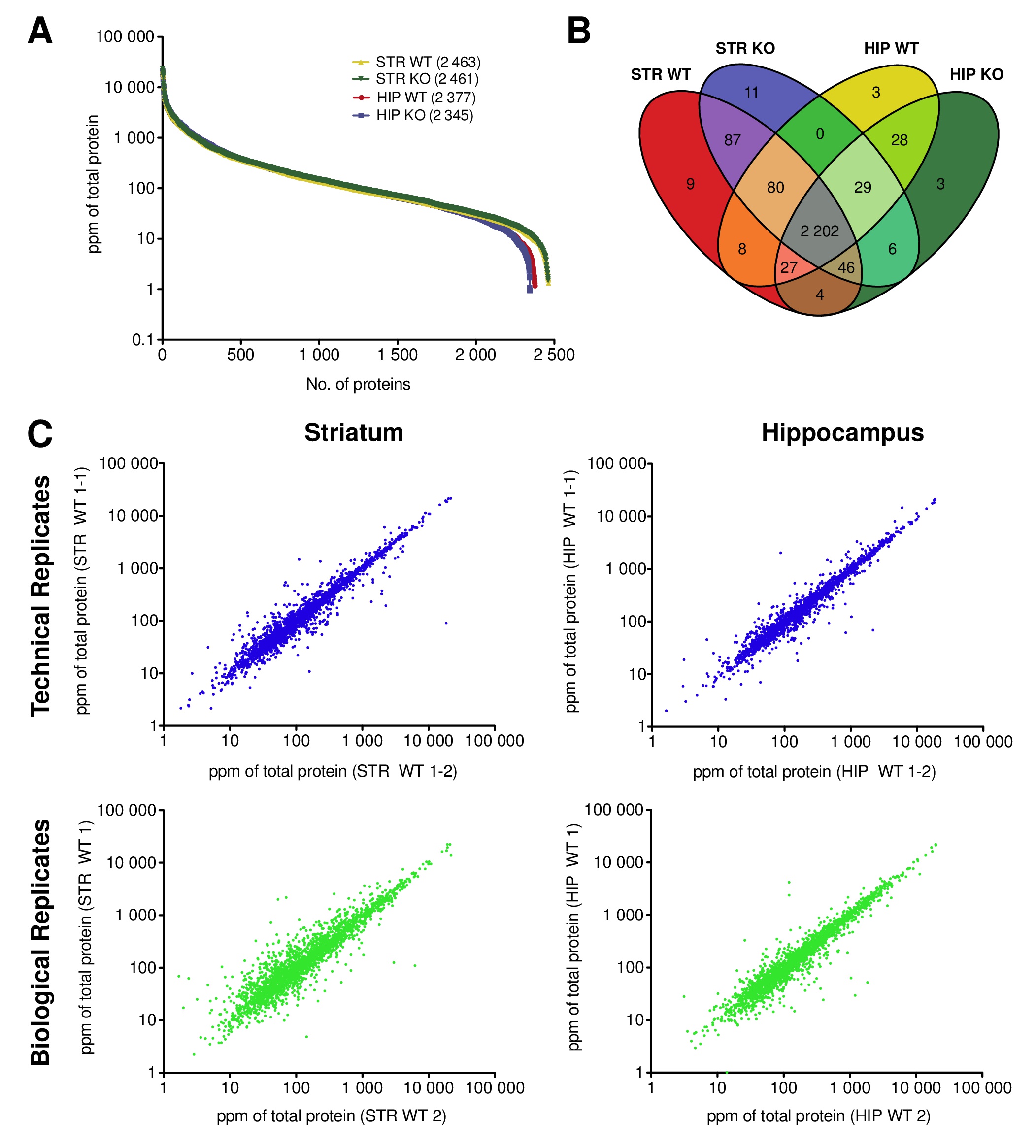

Supplement: FIGURE S2 — Further characterization of the proteomic dataset. (A) Dynamic range of detected PSD proteins in the striatum (STR) and hippocampus (HIP) of WT and Shank3Δ11-/- mutant (KO) mice. (B) Overlap of proteins identified in the PSDs derived from the striatum (STR) and hippocampus (HIP) of WT and Shank3Δ11-/- mutant (KO) mice. Only proteins that have been identified in at least four biological replicates in one condition (either STR WT, STR KO, HIP WT, or HIP KO) were considered. (C) Correlation plots of technical (upper panels) and biological (lower panels) replicates are exemplarily shown for each brain region in WT mice. The correlation plots show high reproducibility of sample preparation and high precision of label-free quantification between technical replicates as well as biological samples. [file Image_2.JPEG]

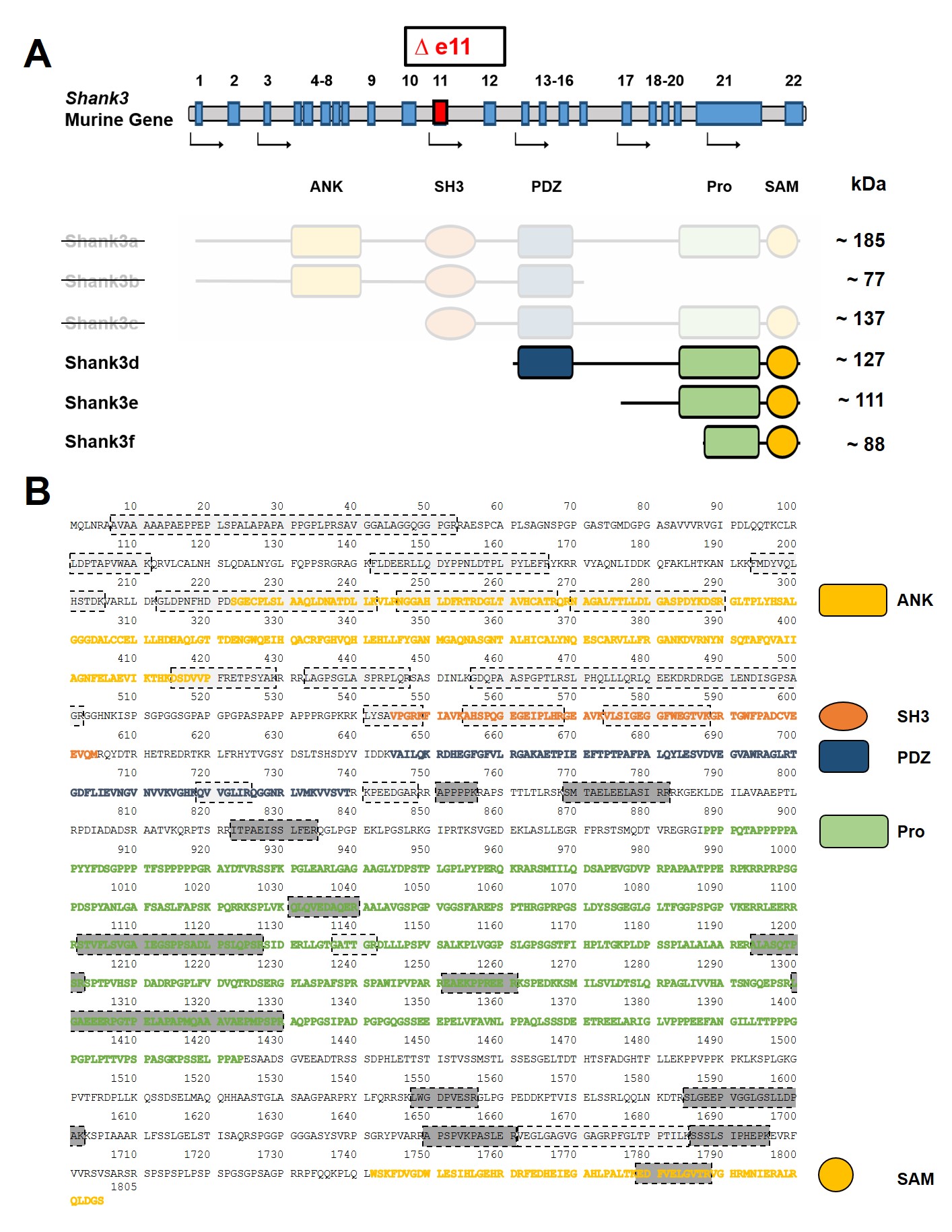

Supplement: FIGURE S3 — Shank3 genetic targeting strategy and resulting pattern of Shank3 isoforms still present in Shank3Δ11-/- mutant mice. (A) Schematic illustration of the murine Shank3 gene (boxes represent respective exons) showing the deletion of exon 11 (indicated in red) and the remaining Shank3 isoforms based on the review by Jiang and Ehlers (2013) excluding alternative splice variants (ANK: N-terminal ankyrin repeats; SH3: Src homology 3 domain; PDZ: PSD95/DLG/ZO-1 domain; Pro: Proline-rich clusters; SAM: sterile alpha motif). Theoretical molecular weight of each Shank3 isoform in kDa was calculated from the isoform specific exon coding sequences and is indicated. (B) Amino acid sequence of the longest Shank3 isoform Shank3a. Colors indicate the respective protein–protein interaction domains; dashed boxes represent the peptides identified by nanoLC–MS, light boxes: detection only in WT tissue, darker boxes filled in gray: detection in both WT and Shank3 mutant tissue. [file Image_3.JPEG]

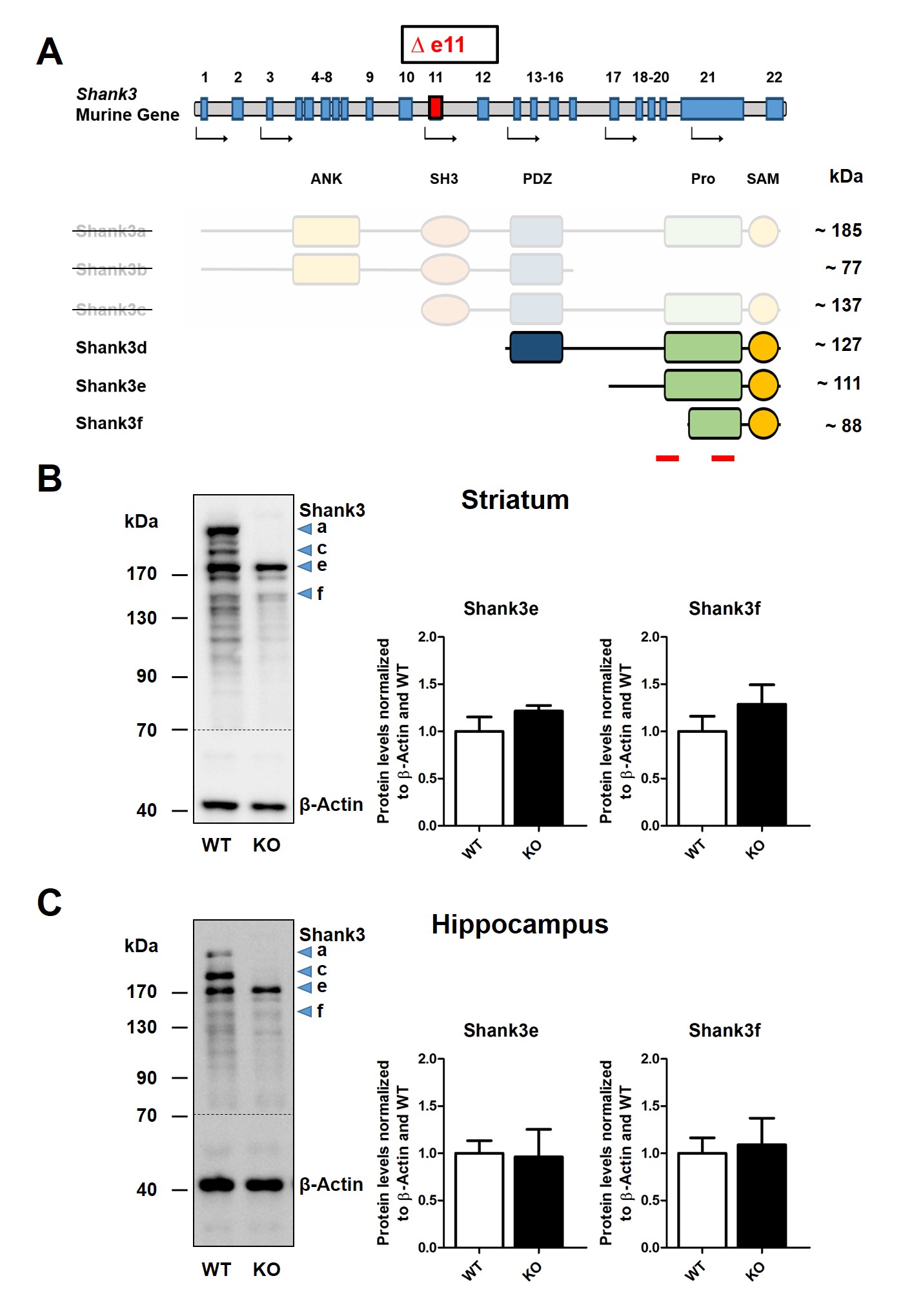

Supplement: FIGURE S4 — Analysis of Shank3 isoforms still present in Shank3Δ11-/- mutant mice. (A) Schematic illustration of the murine Shank3 gene (boxes represent respective exons) showing the deletion of exon 11 (indicated in red) and the remaining Shank3 isoforms based on the review by Jiang and Ehlers (2013) excluding alternative splice variants (ANK: N-terminal ankyrin repeats; SH3: Src homology 3 domain; PDZ: PSD95/DLG/ZO-1 domain; Pro: Proline-rich clusters; SAM: sterile alpha motif). Theoretical molecular weight of each Shank3 isoform in kDa was calculated from the isoform specific exon coding sequences and is indicated. The epitopes of the anti-Shank3 antibody used in this study are marked as red bars below the schematic illustration of the Shank3f isoform (B,C) Western blot analysis of striatal (B) and hippocampal (C) PSD fractions of WT and Shank3Δ11-/- mutant (KO) mice. Shank3 isoforms a, c, e, and f are indicated largely based on the study by Wang et al. (2014). Observed Shank3 isoforms appear with a shift of additional ∼60 kDa from their calculated molecular weight, most probably due to post-translational modifications. Right panel: Analysis of signal intensities for Shank3e and Shank3f, which are still present in the Shank3Δ11-/- mutant PSDs. No significant change was observed. Statistical analysis was performed using an unpaired, two-tailed t-test with a biological sample size of n = 3. [file Image_4.JPEG]
